# Clinical effects of prolonged preoperative fasting in breast cancer surgery: a prospective cohort study

**DOI:** 10.1186/s12893-025-03449-9

**Published:** 2026-01-03

**Authors:** Frida Aretorn, Karin Strigård, Viktor Holmdahl

**Affiliations:** 1 Department of diagnostics and intervention, Umeå University, Umeå, 901 85 Sweden; 2 Department of diagnostics and intervention, University Hospital of Umeå, Umeå, 907 37 Sweden

**Keywords:** Breast cancer, Preoperative care, Fasting, Postoperative complications, Anesthesia, General

## Abstract

**Background:**

Preoperative fasting reduce the risk of pulmonary aspiration during induction of anaesthesia. However, scientific documentation on the correlation between prolonged preoperative fasting and patient discomfort is limited. The aim of the present study was to investigate whether the duration of preoperative fasting correlates with pre- and postoperative patient discomfort, the duration of stay at postoperative care unit and the occurrence of surgical complications within 30 days.

**Methods:**

This prospective observational study included 50 women undergoing elective breast cancer surgery. The Numerical Rating Scale (NRS) was used to assess hunger, thirst, nausea, pain, and anxiety. Patient characteristics, surgical data and occurrence of surgical complications were collected from medical records. Statistical analyses included Sperman’s rank correlation, Pearson’s correlation, Wilcoxon signed rank test, and logistic regression analysis.

**Results:**

The mean fasting duration was 15.1 h for solid food and 12.1 h for fluids. A positive association was observed between fasting duration for solid food and nausea prior to surgery (*p* = 0.067), although not reaching statistical significance. There was no significant correlation between duration of fasting for both solids and fluids and patient discomfort before and after surgery, nor with the duration of stay on the postoperative care unit, nor with the surgical complication rate within 30 days.

**Conclusions:**

No correlation was observed between the duration of preoperative fasting and patient discomfort, the length of stay in the postoperative care unit, or the incidence of surgical complications. Notably, fasting durations highly exceeded the recommended six hours for solids and two hours for clear fluids.

## Introduction

Patient optimisation prior to surgery is a constant topic of research, aiming to enhance postoperative recovery and reduce patient discomfort. An important aspect of this involves reducing the duration of fasting prior to surgery [1].

Preoperative fasting reduces the risk for pulmonary aspiration during induction of general anaesthesia [2]. However, fasting periods exceeding the recommended six hours for solids and two hours for clear fluids are associated with increased patient discomfort including hunger, thirst, pain and nausea. Such discomfort may result in prolonged postoperative hospital stays [3]. Prolonged preoperative fasting is also associated with higher levels of preoperative anxiety [4], a common concern among patients scheduled for surgery. Prior to breast cancer surgery, more than half of the patients experience moderate or high levels of anxiety [5]. To reduce patient discomfort in elective surgery, patients can ingest a clear carbohydrate drink up to two hours prior to induction of anaesthesia [4, 6].

Current guidelines from the European Society of Anaesthesiology recommend a fasting period of two hours for clear fluids and six hours for solid food prior to elective surgery. The guidelines also state that patients should be encouraged to drink until the time recommended [2]. Despite this, patients are frequently prescribed longer fasting than recommended for both fluids and solid food [7, 8]. The actual duration of preoperative fasting is usually unknown, especially in patients undergoing day-case surgery.

Breast cancer surgery is commonly performed as day-case surgery, with reported rates of 72–73% [9, 10]. Since breast cancer is the most common malignancy among women [11, 12], these procedures accounts for a significant proportion of day-case surgeries. Hence, many patients could be affected by a prolonged duration of preoperative fasting and its potentially adverse effects.

While the benefits of preoperative carbohydrate drinks have been demonstrated in various studies, scientific evidence directly linking the duration of preoperative fasting to both subjective perioperative discomfort and postoperative metabolic stress remain limited. The primary aim of this study was to examine whether the length of preoperative fasting correlates with patient-reported discomfort during the perioperative period. Secondary aims were to explore potential associations between fasting duration and the length of stay at the postoperative care unit and the incidence of surgical complications within 30 days.

## Materials and methods

### Study design and setting

The present study was a prospective observational study including women undergoing breast cancer surgery at the University Hospital of Umeå, Sweden. Patients were included between 14th December 2023 and 19th September 2024.

### Study participants

Patients meeting the following inclusion criteria were offered inclusion: women, age 18 years and above, fluent in Swedish and scheduled for breast cancer surgery under general anaesthesia. Exclusion criteria were patients relying on parenteral nutrition or a nasogastric tube habitually or undergoing another surgical procedure simultaneously. Patients were identified from the hospital’s operating schedule and invited to participate after their arrival to their assigned ward.

### Clinical assessments

A Numerical Rating Scale (NRS) ranging from zero to ten was used to assess subjective discomfort. A score of zero indicated no discomfort, whereas a score of ten indicated the strongest imaginable discomfort. Participants were asked to verbally report to the investigating researcher (F.A) the number that best represented the intensity of their sensation. The discomfort variables assessed were hunger, thirst, nausea, pain, and anxiety. Each variable was analysed individually, as well as the total NRS score for all variables. While the NRS is a widely recognised and validated tool for pain assessment [13], it has also been validated for assessment of nausea [14] and anxiety [15].

Assessments were standardised and conducted approximately one hour prior to induction of anaesthesia and one hour after completion of the surgical procedure. The participants also provided information regarding most recent intake of solid food and fluids. Fluid intake was defined as volume greater than 100 ml. According to local protocol, patients were prescribed fasting from midnight for solid food while clear fluids were allowed up to two hours before the scheduled time of induction of anaesthesia.

### Medical record information retrieval

Information on patient age, smoking, comorbidity, and ASA class were collected from the medical records. Data regarding daily medications were also gathered from medical records. Previous experience of postoperative nausea and vomiting as well as potential neoadjuvant chemotherapy were also noted. After discharge, the medical records were evaluated regarding antiemetics and analgesics given peri- and postoperatively, type of breast cancer surgery, type of anaesthesia, duration of stay at the postoperative care unit, potential nerve blockades and operation time. Finally, a review of the medical records was conducted 30 days after surgery to identify surgical complications.

Surgical complications were classed using the Clavien-Dindo classification system, which is commonly used to classify surgical complications. In the present study, surgical complications classified as Clavien-Dindo group II and higher were considered clinically relevant and included in the analysis [16].

### Statistical analysis

All data were gathered in an Access™-database (Microsoft, Redmond, Washington, USA). Statistical analyses were carried out in SPSS v.29.0.2 (IBM Corp., Armonk, NY, USA). Underlying research materials can be accessed after contact with the corresponding author. The duration of preoperative fasting was determined as the interval between the patient’s last reported intake of solids and fluids and the induction of anaesthesia.

The NRS is a numerical scale that should be considered an ordinal scale rather than an interval scale. Hence, non-parametric analyses were performed when analysing data received from the NRS. Spearman’s rank correlation was used to assess correlations between degree of pre- and postoperative discomfort and the duration of preoperative fasting for both solids and fluids. To compare pre- and postoperative NRS scores Wilcoxon signed rank test was used. Pearson’s correlation was used to assess the correlation between length of stay at the postoperative care unit as well as the volume of crystalloid fluids given per hour during anaesthesia and the duration of preoperative fasting. Logistic regression analysis was used to assess the correlation between the frequency of postoperative complications and the duration of preoperative fasting. A P-value < 0.05 was considered statistically significant.

### Sample size

Power was calculated based on a planned comparison between two groups: Patients adhering to the recommended fasting durations (six hours for solids and two hours for liquids) were assumed to have a mean total NRS score of 4 (SD = 2), whereas patients with prolonged fasting were assumed to have a mean score of 6 (SD = 2). Under these assumptions, 16 patients per group (32 in total) would be required to achieve 80% power at a two-sided significance level of 0.05. To account for uncertainty in these estimates, 50 patients were enrolled.

In the actual study, no patient fasted for less than nine hours for solid food, and the planned dichotomous comparison could therefore not be performed. Fasting duration was instead analysed as a continuous variable.

## Results

### Patient characteristics and surgical procedure

Of the 55 patients invited, 50 consented to participate and were enrolled. Patients were included between 14th December 2023 and 19th September 2024. The mean age of the patients was 61.8 years (SD = 14.5), ages ranging from 20 to 87 years.

Regarding surgical procedure, 31 (62%) patients underwent sector resection surgery, 15 (30%) mastectomy, two (4%) underwent bilateral surgery, and two (4%) were operated with re-excision. Most surgeries, 32 out of 50 (64%), were performed as day-case surgery. The mean surgical duration was 1.3 h (SD = 0.5), and the mean duration of anaesthesia was 2.1 h (SD = 0.5). Total intravenous anaesthesia with propofol and remifentanil was administrated to all patients. None of the patients in the present cohort received a nerve blockade. Paracetamol was administered around one hour before surgery. Local anaesthetics (ropivacaine) was infiltrated into the surgical wound at the end of the procedure in 45 patients (90%). Towards the end of surgery, 37 (74%) of the patients received morphine (2–4 g), 30 (60%) patients received parecoxib (40 mg), 22 (44%) received clonidine. No other intraoperative analgesics were used. No anxiolytics were administrated intraoperatively. Regarding airway management, 26 patients (52%) received a laryngeal mask airway during surgery, and 24 patients (48%) received an oral endotracheal tube.

The volume of crystalloid fluids given per hour during anaesthesia showed no correlation with the duration of fasting for fluids (*r*=-0.186, 95% CI: -0.111-0.452, *p* = 0.217). Postoperative analgesics were administrated to 15 (30%) patients prior to postoperative NRS assessments. Postoperative pain management was not standardised in the studied cohort. One (2%) patient received antiemetics prior to the postoperative NRS assessment. Detailed patient characteristics and surgical data are presented in Table 1.

### Antiemetic prophylaxis

Data regarding intraoperative antiemetic prophylaxis are missing from two patients. ondansetron was administered to 46 patients (96%). All received 4 mg except for one patient who received 8 mg. Betamethasone 4 mg was given to 38 patients (79%). Four patients (8%) received droperidol. No additional antiemetic agents were administered. 11 (22%) patients reported previous postoperative nausea and vomiting but received no additional prophylaxis.

**Table 1:** Patient characteristics and surgical data

Total number of participants, *n*	50
Age, mean (SD), years	61.8 (14.5)
Body mass index, mean (SD), kg/m^2^	26.9 (4.5)
ASA* Classification, n (%)	
ASA 1	12 (24.0)
ASA 2	32 (64.0)
ASA 3	6 (12.0)
Diabetes, n (%)	5 (10.0)
Neoadjuvant chemotherapy, n (%)	8 (16.0)
Prophylactic antibiotics, n (%)	18 (36.0)
Previous postoperative nausea and vomiting, n (%)	11 (22.0)
Airway management	
Laryngeal mask airway	26 (52.0)
Oral endotracheal tube	24 (48.0)
Type of surgery, n (%)	
Sector resection and sentinel lymph node biopsy	30 (60.0)
Sector resection and axillary lymph node dissection	1 (2.0)
Bilateral sector resection and bilateral sentinel lymph node biopsy	1 (2.0)
Mastectomy and sentinel lymph node biopsy	11 (22.0)
Mastectomy and axillary lymph node dissection	4 (8.0)
Bilateral mastectomy and bilateral sentinel lymph node biopsy	1 (2.0)
Re-excision without lymph node biopsy	1 (2.0)
Bilateral re-excision without lymph node biopsy	1 (2.0)
Day-case surgery, n (%)	32 (64.0)
Duration of preoperative fasting, mean (SD), hours	
Solid food	15.1 (2.6)
Fluids	12.1 (4.8)
Duration of surgery, mean (SD), hours	1.3 (0.5)
Duration of anesthesia, mean (SD), hours	2.1 (0.5)

*ASA American Society of Anesthesiologists

### Duration of preoperative fasting

**Fig. 1:**
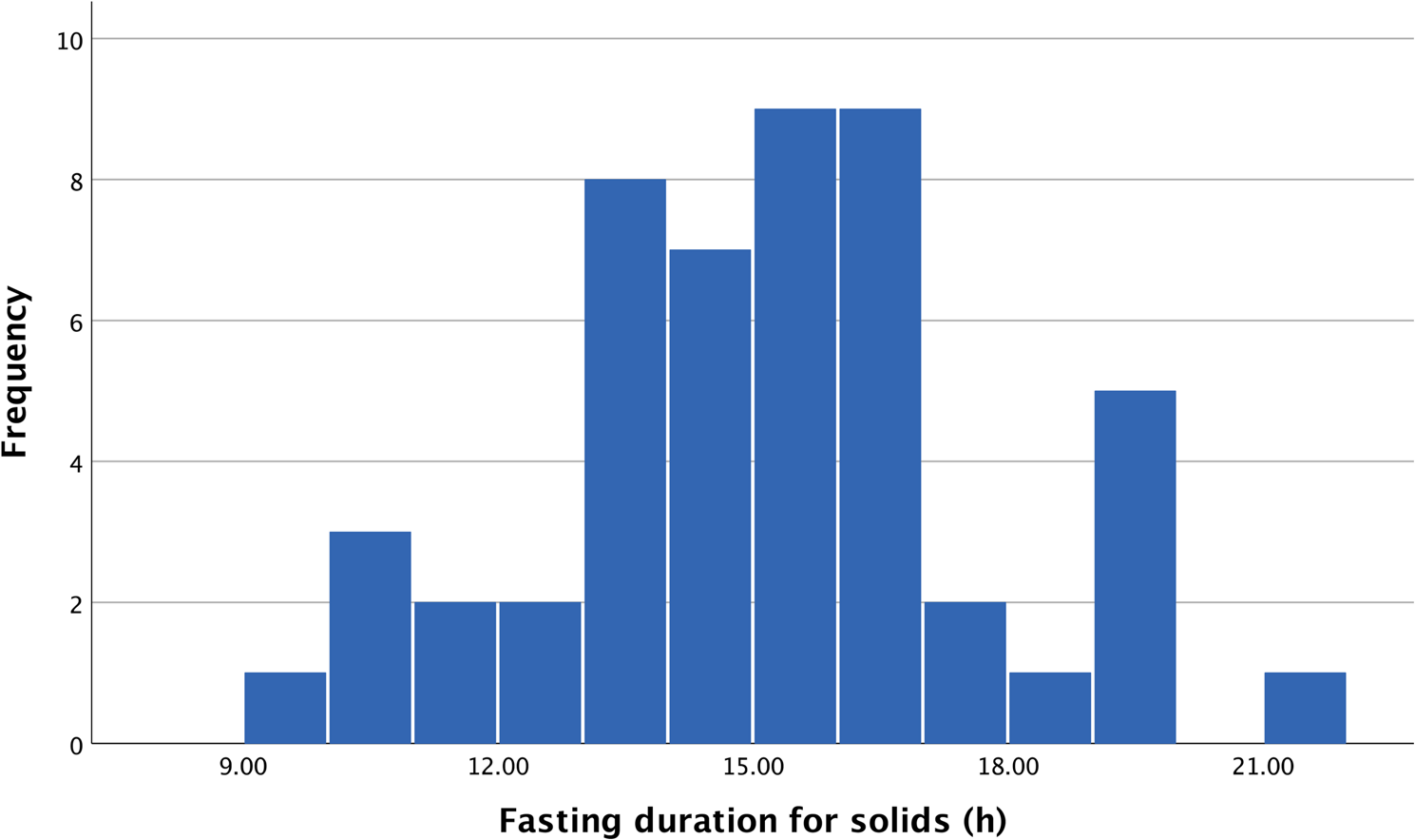
Histogram showing the distribution of the duration of preoperative fasting regarding solid food (hours)

**Fig. 2:**
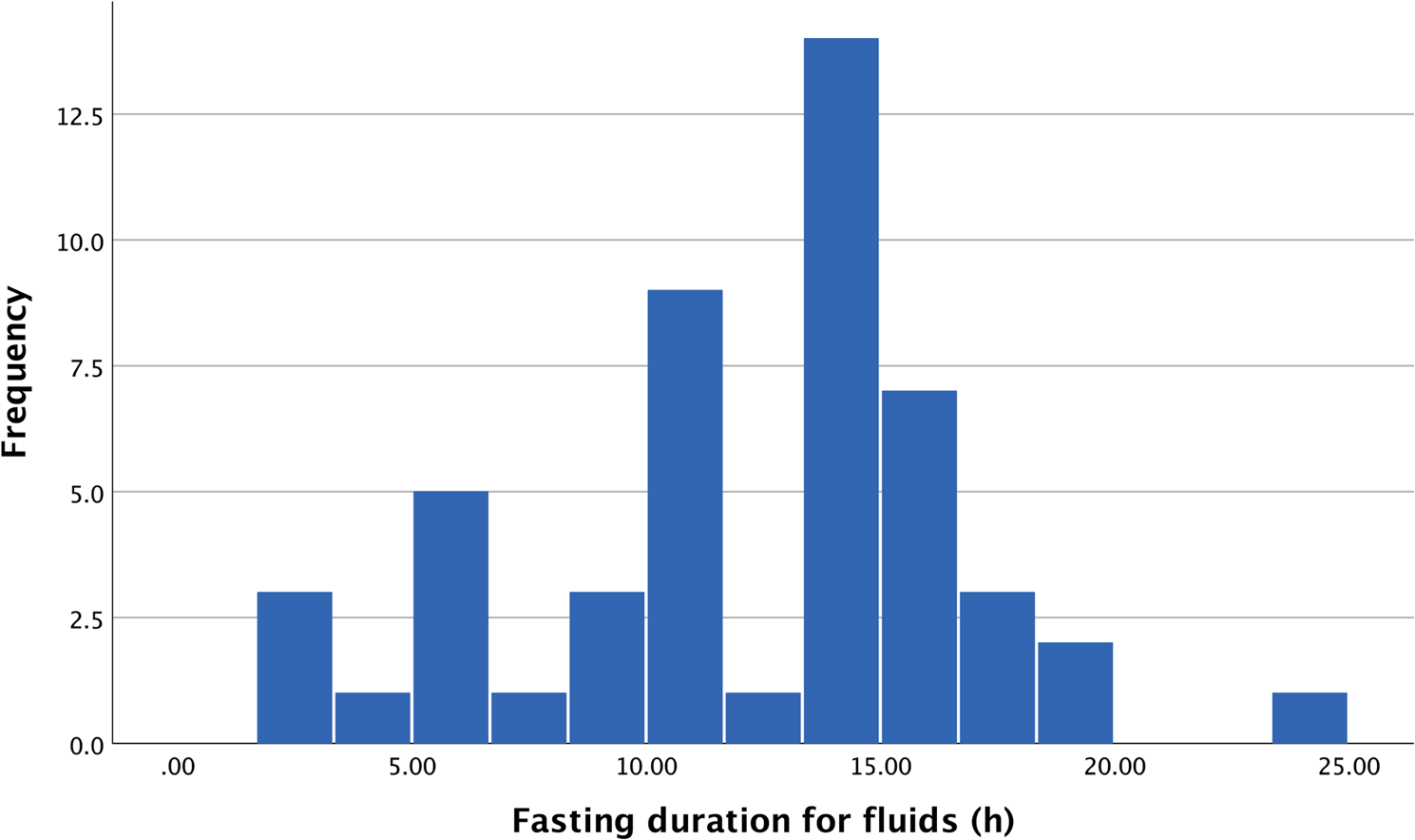
Histogram showing the distribution of the duration of preoperative fasting regarding fluids (hours)

The mean duration of preoperative fasting was 15.1 h (SD = 2.6) for solid food, ranging from 9.6 to 21.2 h (Fig. 1). For fluids the mean duration of preoperative fasting was 12.1 h (SD = 4.8), ranging from 2.0 to 24.2 h (Fig. 2). Notably, a vast majority of the patients, 44 out of 50 (88%), fasted more than 12 h for solid food.

Induction of anaesthesia took place between 08.02 AM and 14.07 PM in the studied cohort.

### Subjective discomfort

Prior to surgery, there was a positive association between the duration of preoperative fasting for solid food and nausea (*r* = 0.261, 95% CI: -0.027-0.509, *p* = 0.067), although not reaching statistical significance. Preoperatively, no correlations were observed between the duration of preoperative fasting for solid food and hunger, thirst, pain, anxiety, and total NRS score (Table 2).

**Fig. 3:**
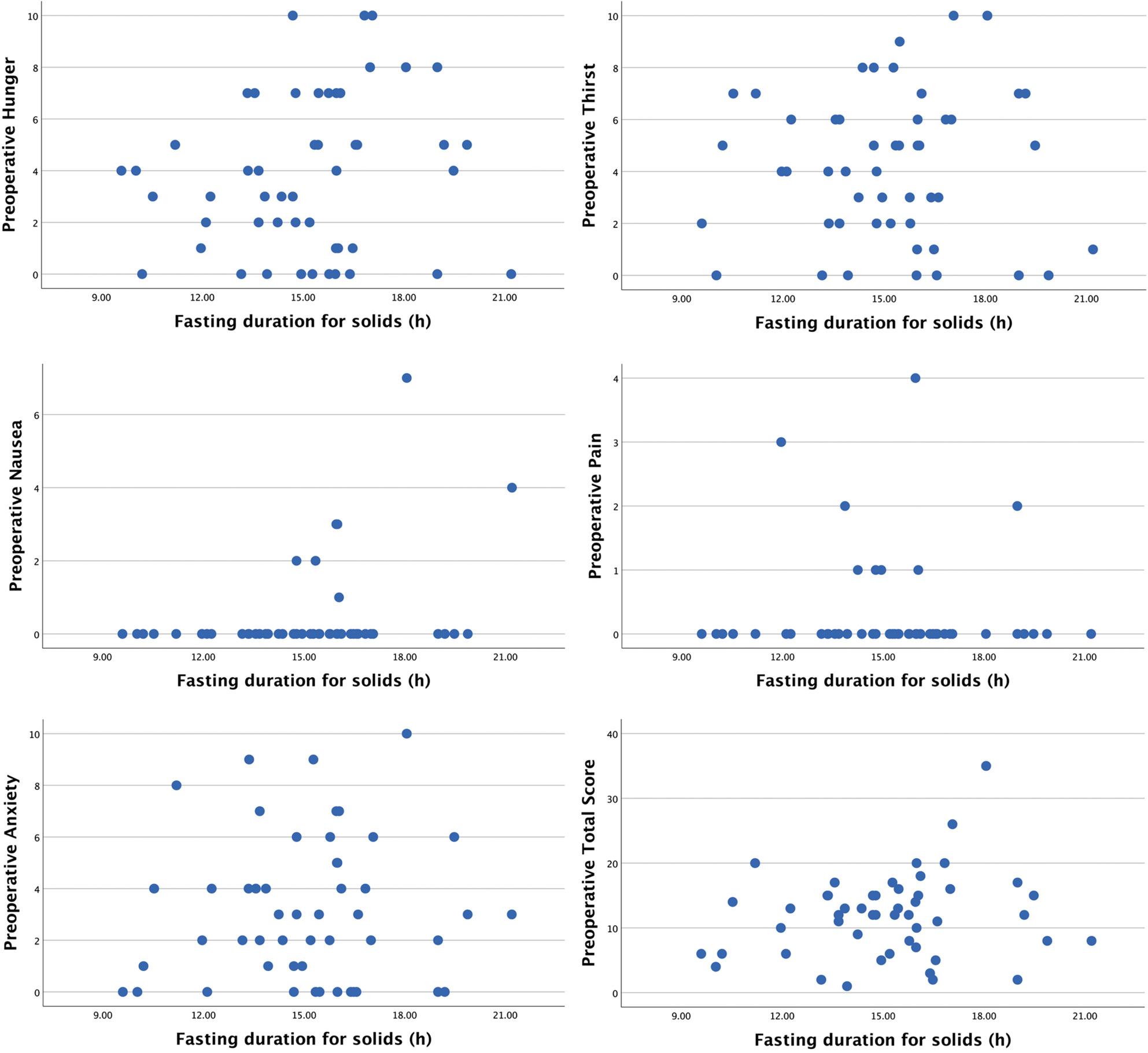
Preoperative NRS-scores regarding hunger, thirst, nausea, pain and anxiety along with a calculated total NRS-score

**Fig. 4:**
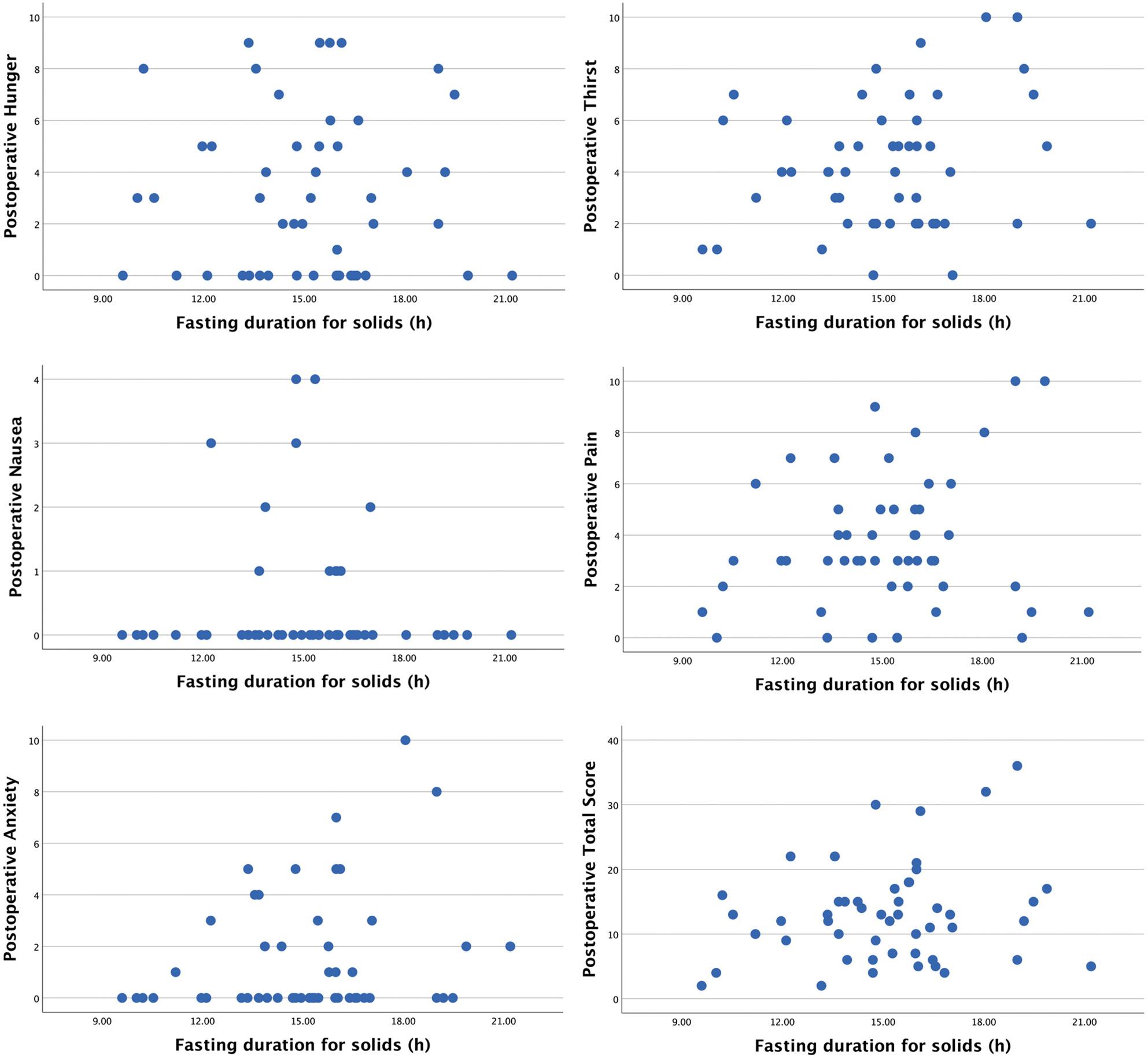
Postoperative NRS-scores regarding hunger, thirst, nausea, pain and anxiety along with a calculated total NRS-score

After surgery, no correlation was observed between duration of preoperative fasting for solid food and hunger, thirst, nausea, pain, anxiety and total NRS score (Table 2). Of note, 39 patients (78%) reported no postoperative nausea. Scatter plots illustrating the relationship between the duration of fasting for solids and pre- and postoperative NRS-assessments are provided in Figs. 3 and 4, respectively.

**Table 2:** Unadjusted correlation between fasting duration for solid food and NRS-assessments

	*r*	95% CI	*P*-Value
Preoperative			
Hunger	0.215	-0.075-0.472	0.133
Thirst	0.041	-0.248-0.323	0.779
Nausea	0.261	-0.027- 0.509	0.067
Pain	-0.041	-0.323-0.248	0.779
Anxiety	0.039	-0.249-0.322	0.785
Total	0.154	-0.139-0.421	0.287
Postoperative			
Hunger	-0.018	-0.302-0.270	0.902
Thirst	0.162	-0.130-0.429	0.260
Nausea	-0.024	-0.308-0.264	0.871
Pain	0.121	-0.171-0.394	0.403
Anxiety	0.179	-0.113-0.442	0.215
Total	0.118	-0.174-0.391	0.415

Regarding duration of preoperative fasting for fluids, no correlation was seen between the duration of fasting and subjective discomfort before nor after surgery.

When comparing preoperative and postoperative NRS-scores, patients experienced a significant increase in pain postoperatively (*p* < 0.001), accompanied by a decrease in anxiety (*p* < 0.001) (Table 3).

**Table 3:** Pre- and postoperative NRS-assessments

	Preoperative NRS-Score		Postoperative NRS-Score		
	Median	IQR	Median	IQR	*P*-value
Hunger	4	6	2.5	5	0.268
Thirst	4	4	4	4	0.484
Nausea	0	0	0	0	0.657
Pain	0	0	3	2	< 0.001
Anxiety	3	4.25	0	2.25	< 0.001
Total	12	8.25	12.5	9.5	0.183

### Length of stay at the postoperative care unit

The mean length of stay at the postoperative care unit was 1.8 h (SD = 0.5, range 0,9–2.8 h). The length of stay at the postoperative care unit showed no correlation with the duration of fasting for solids (*r*=-0.007, 95% CI: -0.291-0.303, *p* = 0.966) nor fluids (*r*=-0.007, 95% CI: -0.290-0.304, *p* = 0.962). Six patients (12%) who were directly transferred from the operating room to the day care unit were excluded from this analysis.

### Surgical complications within 30 days

Surgical complications occurred in nine (18%) out of 50 patients. Observed complications included seroma requiring aspiration in eight patients, hematoma requiring reoperation in one patient and superficial wound infection demanding antibiotics in two patients. There was no correlation between the occurrence of complications and the duration of fasting for solids nor fluids.

## Discussion

### Duration of preoperative fasting

In the present study, the duration of fasting for both solids and fluids were considerably prolonged compared to the recommended six hours for solids and two hours for fluids [2]. The present cohort was prescribed fasting from midnight for solid food accordingly to the local protocol. Patients were allowed to drink clear fluids up to two hours before the scheduled time of induction of anaesthesia. In contrast to the traditional dogma of prescribing patients nil per os (nothing by mouth) from midnight prior to elective surgery [17], both the European and American Societies of Anaesthesiology now emphasises the importance of not keeping patients fasting for longer than recommended [1, 2]. Despite these updated recommendations, our study, in accordance with previous research conducted between 2021 and 2023, reveals the persistence of prolonged fasting durations in patients scheduled for elective surgery [7, 18, 19].

In alignment with this, nutrition is an important component in the well known Enhanced Recovery After Surgery (ERAS) framework, originally developed for colorectal surgery. The ERAS concept includes pre-, peri- and postoperative interventions aiming to enhance postoperative recovery and reduce the risk of complications [20]. However, no standardized ERAS protocol currently exists for breast cancer surgery.

Planning an operating schedule is a delicate work. Allowing and encouraging patients to drink up to two hours prior the induction of anaesthesia would limit flexibility to reschedule or adjust surgical start times.

### Subjective discomfort

The primary aim of our study was to investigate the influence of preoperative fasting duration on patient discomfort. However, our findings revealed no correlation between the duration of preoperative fasting and patient discomfort before and after surgery. Furthermore, differences seem to be small and may be of limited clinical significance. Postoperative increase in pain and decrease in anxiety were anticipated.

Liang et al. demonstrated that fasting for six hours instead of twelve hours for solid food improved patient wellbeing in patients undergoing elective laparoscopic cholecystectomy. The group fasting for six hours reported less hunger and thirst as well as a higher overall comfort score prior to surgery and experienced less pain postoperatively [3]. However, the study by Liang et al. is one of few comparing prolonged fasting with recommended fasting related to patient discomfort.

Several previous studies have investigated the potential benefits of reducing the duration of fasting with preoperative carbohydrate drinks consumed up to two hours prior to induction of anaesthesia. Hausel et al. found that patients who received a carbohydrate drink before elective laparoscopic cholecystectomy or elective colorectal surgery reported less hunger, thirst and anxiety before surgery compared to those fasting from midnight [4]. Similarly, Wang et al. observed that patients who received a carbohydrate drink prior to endoscopic submucosal dissection surgery reported less hunger and thirst both before and after surgery compared to those fasting from midnight [21].

In contrast to these findings, Doo et al. observed no improvement in patient well-being among patients receiving a carbohydrate drink prior to elective thyroidectomy [22]. In alignment with our study, Doo et al. studied a patient group undergoing non-abdominal surgery. It is possible that the duration of fasting has a different impact on patient discomfort depending on the type and duration of the surgery. Unfortunately, Liang et al. did not present the duration of surgery among the studied patients and Hausel et al. only assessed patient discomfort prior to surgery.

### Length of stay at the postoperative care unit

In the present study, we observed no correlation between the duration of preoperative fasting for solids and fluids, and the length of stay at the postoperative care unit. Previous research investigating the impact of fasting time on length of hospital stay have presented inconclusive results. Liang et al. observed shorter duration of hospital stay in the research group fasting for six hours compared to the control group fasting for twelve hours among patients undergoing laparoscopic cholecystectomy [3]. Conversely, neither Mathur et al. nor Sada et al. observed shorter duration of hospital stay when shortening the duration of fasting with a preoperative carbohydrate drink [23, 24].

In contrast to previous conducted research, we chose to analyse length of stay at post anaesthesia care unit rather than total length of stay at hospital. In the studied cohort, the duration of stay at the postoperative care unit was primarily determined by patient discomfort, including degree of nausea and pain. Conversely, the time of discharge from the hospital was more often determined by logistics such as distance between hospital and home. The present study was conducted in the county of Västerbotten where distances can be up to 360 km. However, the length of stay at the postoperative care unit at the present hospital is also, to some extent, determined by logistical factors such as availability of transport.

### Surgical complications within 30 days

Surgical complications within 30 days did not correlate with the duration of fasting for solids nor fluids.

Insulin resistance is a central response to surgical trauma [25] and risk factor for postoperative complications. In a study from 2010, Sato et al. showed that insulin resistance is associated with an increased risk of postoperative major complications in patients undergoing cardiac surgery [26]. The use of carbohydrate drinks to shorten the duration of preoperative fasting has been proposed as a strategy to reduce postoperative insulin resistance. However, findings across various studies remain inconclusive [27–30].

Important to note is that the degree of postoperative insulin resistance is related to the extent of the surgical procedure, with larger procedures yielding greater degrees of insulin resistance [25]. Given that breast cancer surgery is a relatively minor surgical procedure, one would expect the degree of insulin resistance following breast cancer surgery to be modest. Consequently, the potential reduction in insulin resistance given by a shortened duration of preoperative fasting would be less pronounced compared to more extensive procedures.

### Study limitations and strengths

In the present study the fasting durations for solid food are highly concentrated around 15 h. None of the patients fasted for shorter than nine hours for solids, and that the vast majority, 44 out of 50 (88%) patients, had a duration of fasting exceeding twelve hours for solids. Hence, there is a complete lack of patient fasting for the recommended durations of six hours for solids. This narrow distribution limits the ability to find relevant correlations. A wider distribution in fasting durations would therefore have been beneficial for detecting potential correlations, as well as acting as a desired reference. Hence, it remains possible that adhering to the recommended fasting durations could result in less patient discomfort before and after surgery.

NRS is useful in many settings, but it is important to acknowledge that it has not been validated when assessing hunger and thirst. Evaluating hunger is difficult due to the range of manifestations including stomach discomfort, poor concentration, irritability etc. These diverse expressions of hunger could potentially complicate using the NRS for hunger assessment. The postoperative NRS-assessments could also be affected by administrations of postoperative analgesics and antiemetics prior to the assessments took place. Since latest intake of solid food and fluids is self-reported, the risk of recall bias must be considered. However, all patients where asked this question by the same researcher in a standardized manner.

To the best of our knowledge, this is the first study on the impact of duration of fasting on patient discomfort, the length of stay at the postoperative care unit, and postoperative complications in breast cancer surgery. As the present study is a prospective observational study, the results are expected to be well representative of women undergoing breast cancer surgery. However, as with all observational studies, the findings should be interpreted in the context of potential uncontrolled biases. No patients were lost to follow up in the present cohort. Moreover, we believe that the results from the study are likely to be appliable to other patient groups undergoing soft tissue procedures with comparable surgical duration. However, since the study only included women, this must be considered when applying the results to other patient groups.

## Conclusion

In the studied cohort, the mean duration of preoperative fasting was 15 h for solid food and 12 h for liquids - substantially longer than the durations recommended by the American and European Societies of Anaesthesiology. We found no correlation between the duration of preoperative fasting and patient discomfort before and after surgery, length of stay in the postoperative care unit, or the incidence of surgical complications within 30 days. However, because no participant fasted for fewer than 9 h for solids, that the potential benefits of adhering to guideline-recommended fasting durations compared with prolonged fasting cannot be excluded. Further studies are needed to determine whether a recommended duration of fasting confer advantages over prolonged fasting for both solids and liquids in patients undergoing breast cancer surgery.

## Data Availability

The data that support the findings of this study are available from the corresponding author upon reasonable request.

## Abbreviations

ASA: American Society of Anesthesiologists
ERAS: Enhanced Recovery After Surgery
NRS: Numerical Rating Scale
